# Percutaneous nephrostomy versus retrograde ureteral stenting for acute upper obstructive uropathy: a systematic review and meta-analysis

**DOI:** 10.1038/s41598-021-86136-y

**Published:** 2021-03-23

**Authors:** Ismail Zul Khairul Azwadi, Mohd Noor Norhayati, Mohd Shafie Abdullah

**Affiliations:** 1 Department of Radiology, School of Medical Sciences, Universiti Sains Malaysia, Kubang Kerian, Malaysia; 2 Department of Family Medicine, School of Medical Sciences, Universiti Sains Malaysia, Kubang Kerian, Malaysia; 3 Hospital Universiti Sains Malaysia, Universiti Sains Malaysia Health Campus, Jalan Raja Perempuan Zainab 2, 16150 Kota Bharu, Kelantan Malaysia

**Keywords:** Nephrology, Urology, Kidney diseases

## Abstract

Acute obstructive uropathy is associated with significant morbidity among patients with any condition that leads to urinary tract obstruction. Immediate urinary diversion is necessary to prevent further damage to the kidneys. In many centres, the two main treatment options include percutaneous nephrostomy (PCN) and retrograde ureteral stenting (RUS). The purpose of this study if to compare the efficacy and safety of PCN and RUS for the treatment of acute obstructive uropathy. We searched the Cochrane Central Register of Controlled Trials (CENTRAL), MEDLINE, CINAHL, EMBASE, the World Health Organisation International Clinical Trials Registry Platform and ClinicalTrials.gov. We also searched the reference lists of included studies to identify any additional trials. We included randomised controlled trials and controlled clinical trials comparing the outcomes of clinical improvement (septic parameters), hospitalisation duration, quality of life, urinary-related symptoms, failure rates, post-procedural pain [measured using a visual analogue scale (VAS)] and analgesics use. We conducted statistical analyses using random effects models and expressed the results as risk ratio (RR) and risk difference (RD) for dichotomous outcomes and mean difference (MD) for continuous outcomes, with 95% confidence intervals (CIs). Seven trials were identified that included 667 patients. Meta-analysis of the data revealed no difference in the two methods in improvement of septic parameters, quality of life, failure rates, post-procedural pain (VAS), or analgesics use. Patients receiving PCN had lower rates of haematuria and dysuria post-operatively and longer hospitalisation duration than those receiving RUS. PCN and RUS are effective for the decompression of an obstructed urinary system, with no significant difference in most outcomes. However, PCN is preferable to RUS because of its reduced impact on the patient’s post-operative quality of life due to haematuria and dysuria, although it is associated with slightly longer hospitalisation duration.

## Introduction

Obstructive uropathy is one of the most common conditions affecting the urinary system and is a significant cause of renal impairment, leading to end-stage renal failure. It is a condition wherein impedance of urinary flow causes dilatation of the pelvicalyceal system, resulting in damage to the renal parenchyma^1^; 9.2% of chronic kidney disease cases are caused by obstruction of the urinary tract^2^. No or suboptimal treatment will lead to inevitable permanent chronic kidney disease through a combination of ischaemic or disuse-induced tubular injury, inflammation and interstitial renal fibrosis^3,4^.

Urgent decompression is warranted in cases of acute obstructive uropathy, either percutaneously via a nephrostomy tube or retrogradely via ureteral stent placement. This decompression prevents further worsening of renal function, inflammation and ischaemia to renal parenchyma that can eventually progress to irreversible chronic kidney disease.

The choice of technique depends on operator preference, availability of interventional radiologists or urologists and the centre’s standard protocol. At our institution, PCN is the preferred approach of urinary diversion for acute obstructive uropathy due to better availability of interventional radiologists and it is a much cheaper procedure as most of our patients are from lower income group.

Currently, there are no standard guidelines on the preferred method of urgent urinary tract decompression in the setting of acute obstructive uropathy. There is conflicting evidence in the literature regarding these two methods. PCN is associated with higher rates of sepsis and mortality compared to RUS^5^. Other outcomes such as time to definitive stone management, rates of spontaneous stone passage and initiation of stone metabolic workup were not found to be statistically different^6^. Therefore, an evidence-based comparison based on pooled data from available clinical trials comparing these two approaches is warranted to provide a potential basis for establishing a standard guideline in the management of acute upper obstructive uropathy. The objective of this study was to compare the efficacy and safety of PCN and RUS in treating this condition.

## Results

### Search results

**Figure 1:**
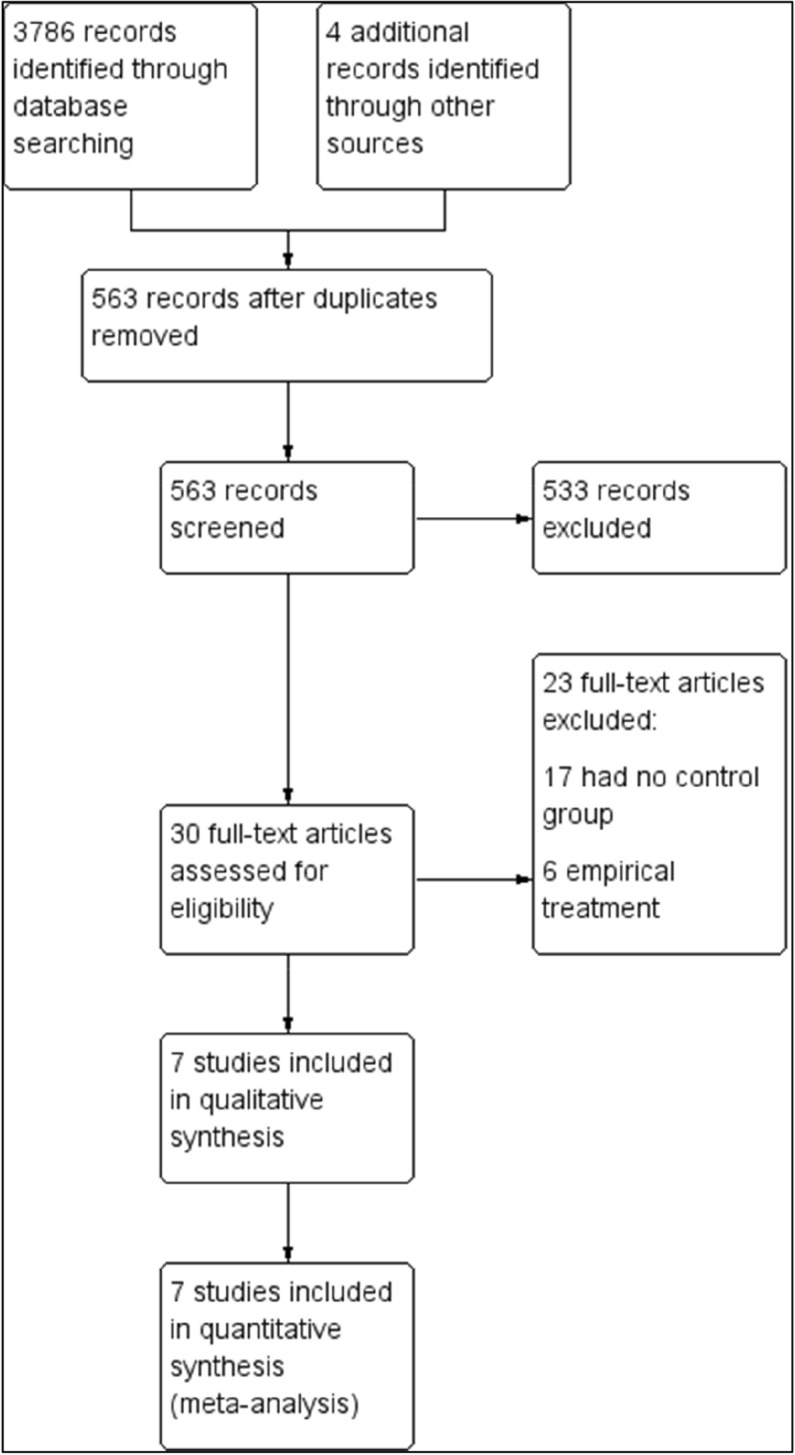
Study flow diagram.

We retrieved 3786 records from the search databases and four additional records from other sources (Fig. 1). In total, 563 records were screened after removing duplicates. We reviewed 30 full-text articles and excluded 23 articles, of which seven were non-comparative trials and six investigated empirical treatment. Empirical treatment refers to the use of PCN or RUS as preventive measures in conditions wherein obstructive uropathy is anticipated but has not yet occurred.

### Included studies

We included seven trials (N = 667)^7–14^. These trials are summarized in Table 1.

**Table 1:** Summary of included studies in meta-analysis.

Author, year	Location	Age (years)	Settings	Recruitment	Study design	Number of participants, n	Interventions	Outcomes
Ahmad, 2013	Bahawalpur, Pakistan	PCN: 43 ± 9.65	Single centre	Referral to Urology	RCT	300	PCN, n = 200	Failure rates Haematuria
		RUS: 40 ± 10.35					RUS, n = 100	
de Sousa, 2018	Braga, Portugal	PCN: 63.1	Single centre	Presented at ED	CCT	50	PCN, n = 18	Mobility Self-care Usual activity Pain/discomfort Anxiety/depression Haematuria Dysuria Urgency Frequency Use of analgesics
		RUS: 54.5					RUS, n = 32	
Joshi, 2001	Bristol, UK	PCN: 56 ± 9	Single centre	Referral to stone management unit	CCT	34	PCN, n = 13	Mobility Self-care Usual activity Pain/discomfort Anxiety/depression Haematuria Dysuria Urgency Frequency Use of analgesics
		DJS: 55 ± 14					DJS, n = 21	
Mokhmalji, 2001	Aleppo, Syria and Mannheim, Germany	PCN: 49	Multicentre	Referral to Urology	RCT	40	PCN, n = 24	Failure rates Use of analgesics
		RUS: 55					RUS, n = 16	
Pearle, 1998	Texas, USA	PCN: 41.3 ± 13.0	Single centre	Presented at ED	CCT	42	PCN, n = 21	Failure rates Use of analgesics Normalization for WBC normalization Duration to defervescence Duration of hospitalization Pain post procedure
		RUS: 41.3 ± 14.5					RUS, n = 21	
Shoshany, 2019	Tel Aviv, Israel	PCN: 54 (46.5–61)	Multicentre	Referral to Urology	CCT	75	PCN, n = 30	Mobility Self-care Usual activity Pain/discomfort Anxiety/depression Haematuria Use of analgesics Pain post procedure
		DJS: 55 (39.5–70.5)					DJS, n = 45	
Wang, 2015	Taiwan	PCN: 58.21 ± 10.89	Single centre	Referral to Urology	CCT	107	PCN, n = 53	Duration to defervescence Duration for WBC normalization Duration of hospitalization
		RUS: 57.52 ± 11.93					RUS, n = 54	

### Participants

All seven trials were conducted in healthcare settings. The total number of participants was 667. Four of the trials were conducted in the urology departments from referral cases^9,10,12,14^, two trials included patients who presented to the emergency department^7,13^ and one trial included patients referred to a stone management unit^8^. One multicentre trial was conducted in urology units in Germany and Syria^9^. Two trials were RCTs^9,10^, whereas five were CCTs^7,8,12–14^.

### Intervention

All trials compared PCN and RUS as the intervention and control groups, respectively. In all trials, the procedures were conducted by urologists. Percutaneous nephrostomies were conducted under ultrasound guidance with local anaesthesia. Retrograde ureteral stents were inserted using a cystoscope after patients were put under general anaesthesia. All stents were double-J stents.

### Outcomes

Two trials analysed parameters of clinical success, which included septic parameters and hospitalisation duration in patients with obstructive uropathy^7,12^. Three trials reported patients’ quality of life and urinary-related symptoms^8,13,14^. For the secondary outcomes, three trials reported failure rates^7,9,10^, two trials reported post-procedural pain^7,14^ and four trials reported analgesics use^8,9,13,14^.

All trials that reported quality of life used a similar questionnaire, that is, the EuroQol EQ-5D-3L^8,14,15^, which is the three-level version of the EQ-5D. This measure includes a descriptive system that contains five dimensions: mobility, self-care, usual activities, pain/discomfort and anxiety/depression. Each dimension has three levels: no problems, some problems and extreme problems. Participants were asked to express their health state for the most appropriate statement in each of the five dimensions.

### Excluded studies

We excluded 13 trials. Seven trials had no control group^16–22^. Six trials analysed PCN and RUS as empirical treatment^23–28^.

### Risk of bias in included studies

**Figure 2:**
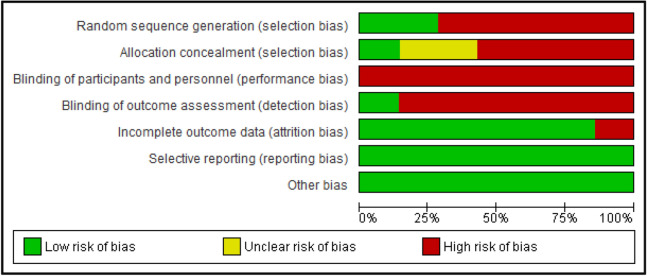
Risk of bias graph: review authors’ judgements about each risk of bias item presented as percentages across all included studies.

**Figure 3:**
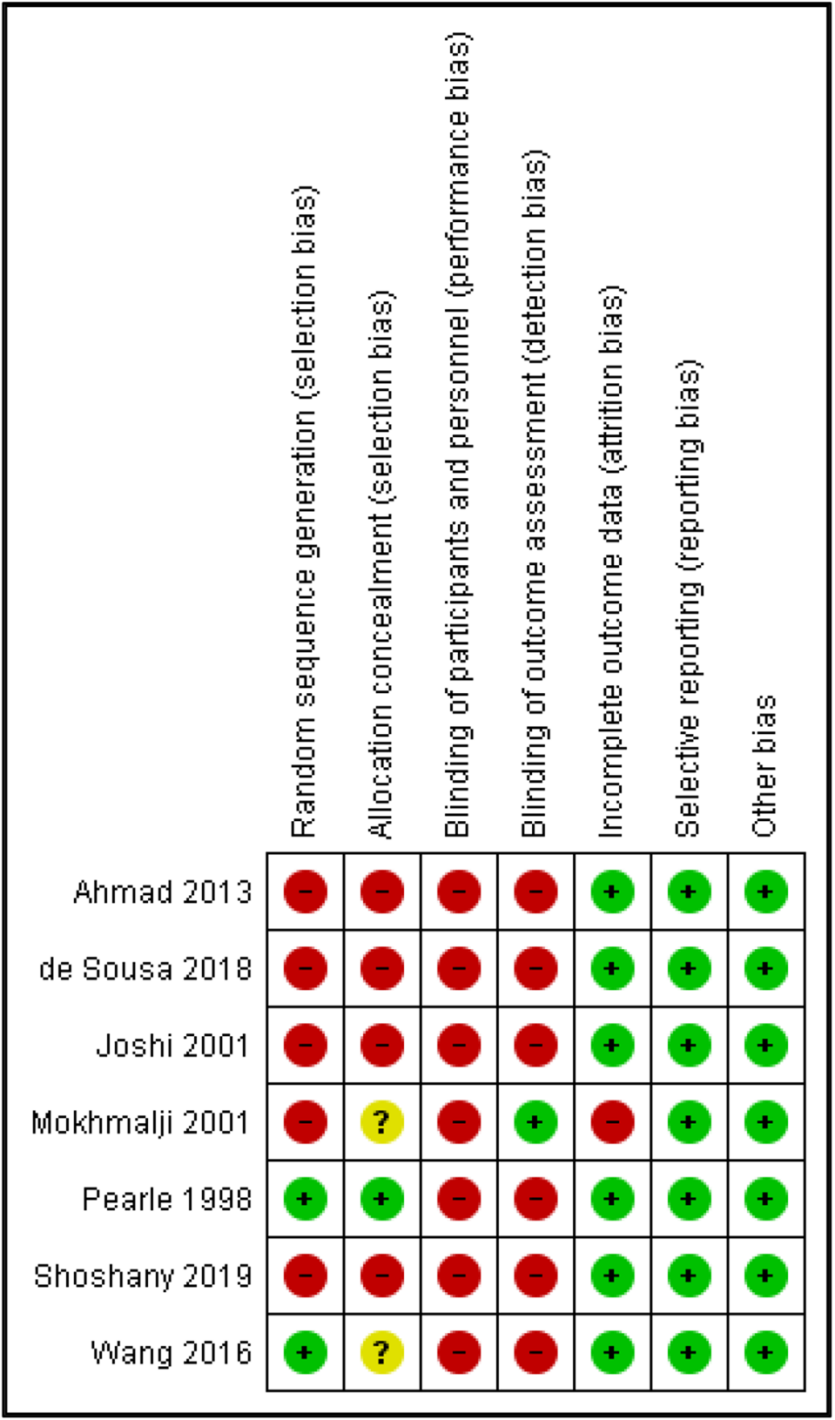
Risk of bias summary: review authors’ judgements about each risk of bias item for each included study.

The risk of bias assessment is shown in Figs. 2 and 3. The proportion of the assessment of included studies based on the risk of bias for each ‘risk of bias’ indicator is shown in Fig. 2. The risk of bias analysis for the individual studies is shown in Fig. 3.

### Allocation

Three trials reported the use of the random number tables method of randomisation^7,10,12^. One trial used quasi-randomisation based on odd- or even-numbered birth year^29^. The remaining three trials did not use any randomisation; patients were allocated based on the preference of the performing surgeon, who was not aware of the trials^8,14,15^.

### Blinding

Blinding of participants and personnel was not feasible because all trials involved a procedure.

### Incomplete outcome data

In one trial, RUS insertion failure occurred in three patients who were then subjected to PCN as the urinary diversion method. These patients were still included in the analysis in the RUS group^10^. In another trial, four of 20 patients in the RUS group had an unsuccessful procedure and thus were subjected to PCN^9^. The four patients were analysed as a separate group; the outcomes in the RUS group were analysed based on the remaining 16 patients. In one trial, two patients were missing from both the PCN and RUS groups because of loss to follow-up^12^. These patients were not included in the final analysis. In one trial, four patients were missing for various reasons in each group^11^. They were included in the analysis of outcomes.

### Selective reporting

All seven trials reported outcomes based on the objectives and measured in the methods^7–14^.

### Other potential sources of bias

There were no other potential sources of bias in the included studies.

### Effects of interventions

#### Primary outcomes

**Figure 4:**
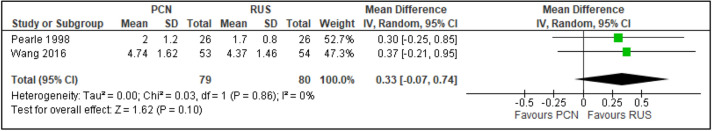
Comparison between PCN and RUS for outcome duration to WBC normalisation.

**Figure 5:**
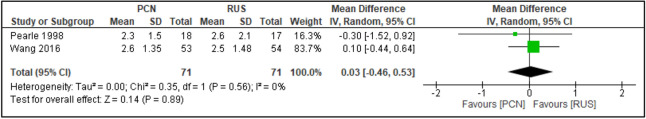
Comparison between PCN and RUS for outcome duration to defervescence.

**Figure 6:**
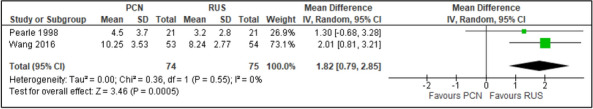
Comparison between PCN and RUS for outcome hospitalisation duration.

**Figure 7:**
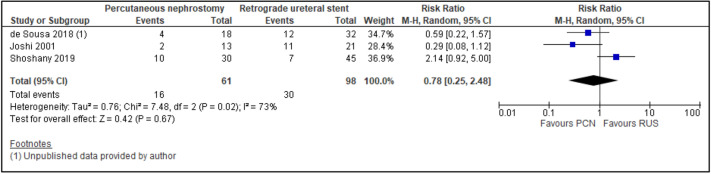
Comparison between PCN and RUS for outcome QoL – mobility.

**Figure 8:**
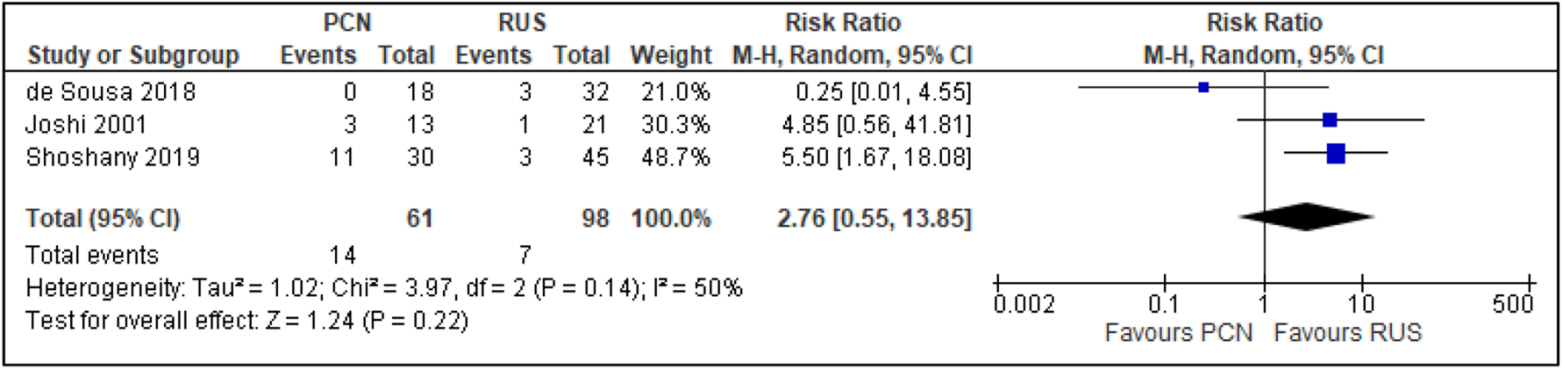
Comparison between PCN and RUS for outcome QoL – self-care.

**Figure 9:**
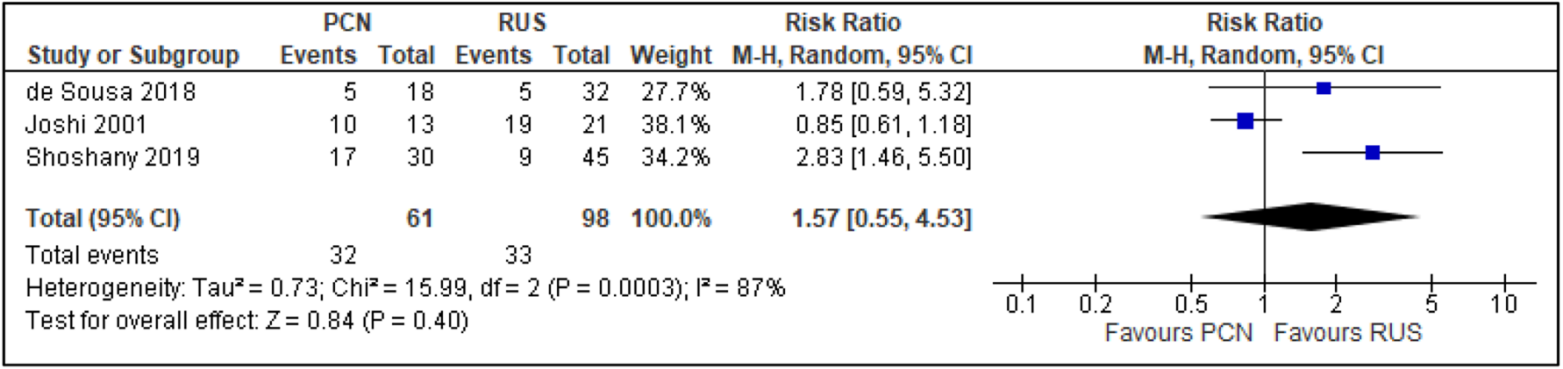
Comparison between PCN and RUS for outcome QoL – usual activity.

**Figure 10:**
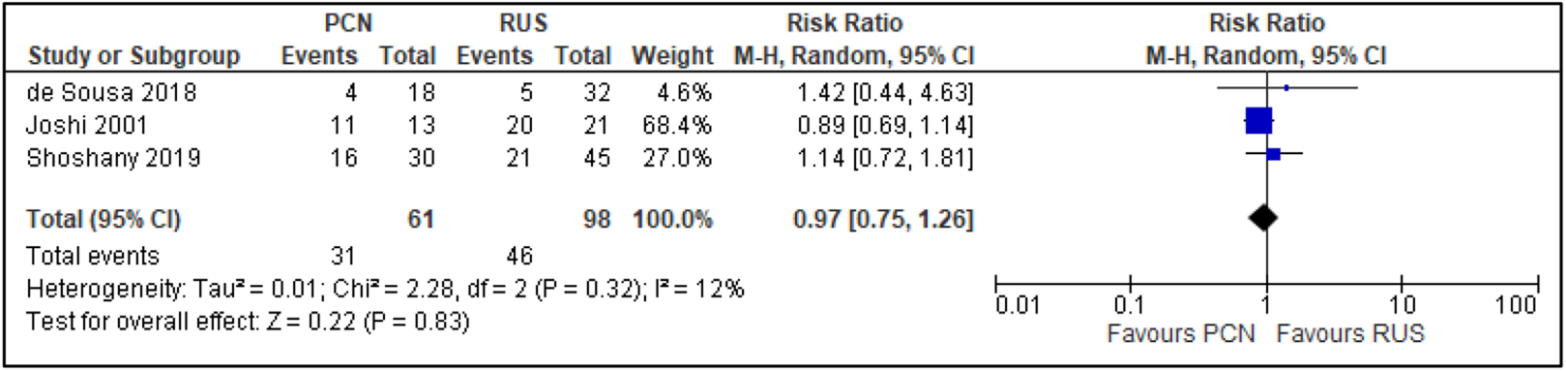
Comparison between PCN and RUS for outcome QoL – pain/discomfort.

**Figure 11:**
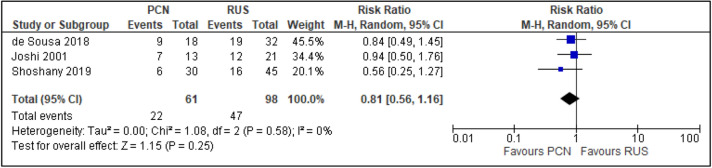
Comparison between PCN and RUS for outcome QoL – anxiety/depression.

**Figure 12:**
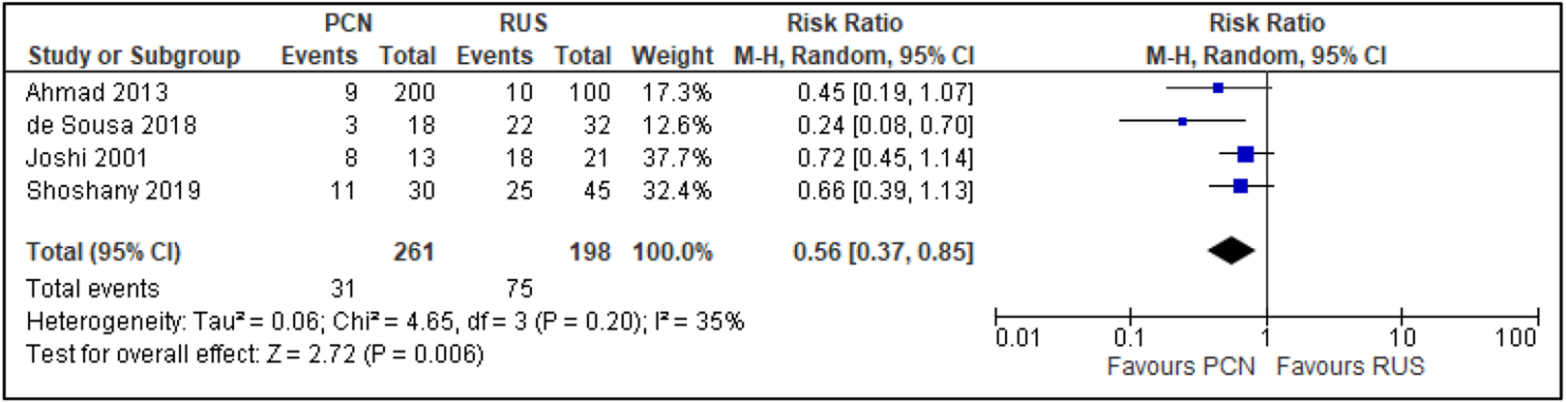
Comparison between PCN and RUS for outcome haematuria.

**Figure 13:**
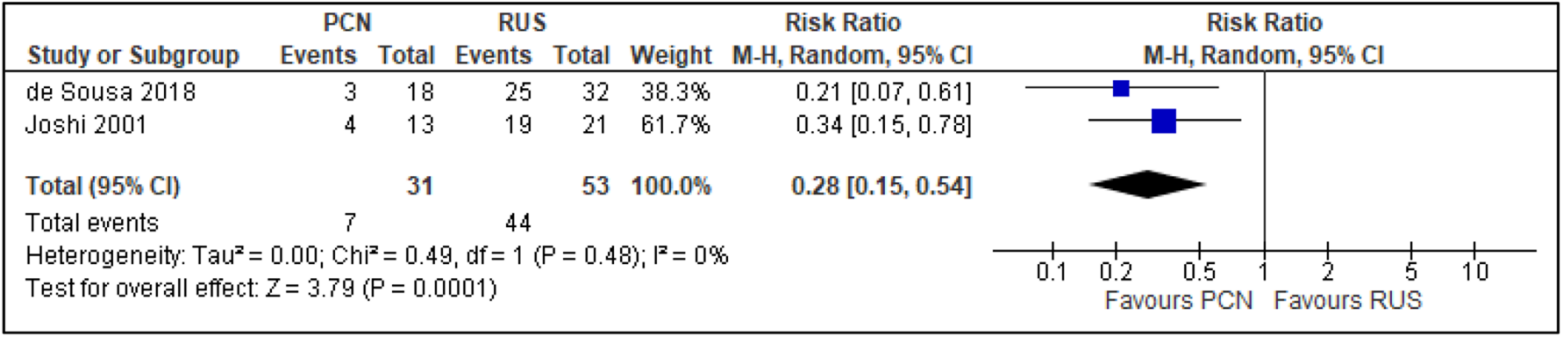
Comparison between PCN and RUS for outcome dysuria.

**Figure 14:**
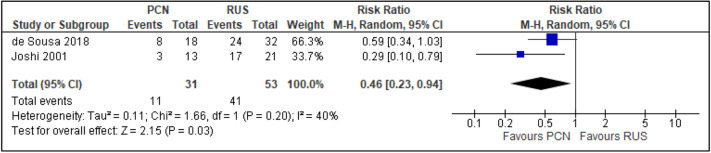
Comparison between PCN and RUS for outcome frequency.

**Figure 15:**
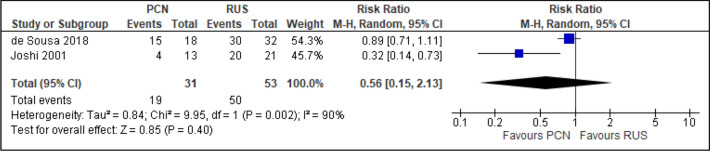
Comparison between PCN and RUS for outcome urgency.

All trials reported the primary outcomes of this meta-analysis^7–14^:

- Clinical success of PCN: Three trials reported the clinical success outcomes regarding improvement in septic parameters^7,12,14^.
  - Improvement in septic parameters:
    i. WBC normalisation duration: Two trials compared the WBC normalisation duration (days) post-operatively^7,12^ (two trials; 149 participants; MD [95% C1] 0.33 [− 0.07 to 0.74]; I^2^ = 0%; *P* = 0.100; moderate-quality evidence; Fig. 4; Table 2).
    ii. Duration to defervescence. Three trials reported the duration to defervescence^7,12,14^; however, one trial reported the median duration (interquartile range [IQR]) and thus was not included in the meta-analysis^14^ (two trials; 142 participants; MD [95% CI] 0.33 [− 0.46 to 0.53]; I^2^ = 0%; *P* = 0.890; moderate-quality evidence; Fig. 5; Table 2).
- Hospitalisation duration: Three trials reported the hospitalisation duration^7,12,14^; however, one trial reported the findings as median (IQR) and thus was not included in the meta-analysis^14^ (two trials; 149 participants; MD [95% CI] 1.82 [0.79 to 2.85]; I^2^ = 0%; *P* < 0.001; moderate-quality evidence; Fig. 6; Table 2).
- Quality of life: Three trials reported patient quality of life^8,14,15^. All trials had a high risk of random sequence generation and allocation concealment.
- Mobility: Three trials reported quality of life regarding mobility^8,14,15^ (RR [95% CI] 0.78 [0.25 to 2.48]; RD [95% CI] − 0.10 [− 0.42 to 0.22]; I^2^ = 73%; *P* = 0.670; very low-quality evidence; Fig. 7; Table 2).
- Self-care: Three trials reported quality of life regarding self-care^8,14,15^ (RR [95% CI] 2.76 [0.55 to 13.85]; RD [95% CI] 0.12 (− 0.18 to 0.43]; I^2^ = 50%; *P* = 0.220; Fig. 8).
- Usual activity: Three trials reported quality of life regarding usual activity^8,14,15^. (RR [95% CI] 1.57 [0.55 to 4.53]; RD [95% CI] 0.13 [− 0.16 to 0.41]; I^2^ = 87%; *P* = 0.400; very low-quality evidence; Fig. 9; Table 2).
- Pain/discomfort: Three trials reported quality of life regarding pain or discomfort^8,14,15^ (RR [95% CI] 0.97 [0.75 to 1.26]; RD [95% CI] 0.00 [− 0.13 to 0.13]; I^2^ = 12%; *P* = 0.830; low-quality evidence; Fig. 10; Table 2).
- Anxiety/depression: Three trials reported quality of life regarding anxiety or depression^8,14,15^ (RR [95% CI] 0.81 [0.56 to 1.16]; RD [95% CI] − 0.12 [− 0.26 to 0.03]; I^2^ = 0%; *P* = 0.250; Fig. 11).
- Urinary-related symptoms:
- Haematuria: Four trials reported haematuria post-procedure^8,10,14,15^. All trials had high risk of random sequence generation and allocation concealment (RR [95% CI] 0.56 [0.37 to 0.85]; RD [95% CI] − 0.24 [− 0.47 to − 0.01]; I^2^ = 35%; *P* < 0.001; very low-quality evidence; Fig. 12; Table 2).
- Dysuria: Two trials reported dysuria^8,15^ (RR 0.28, 95% CI 0.15 to 0.54; RD − 0.61, 95% CI − 0.78 to − 0.43; I^2^ = 0%; *P* < 0.001; moderate-quality evidence; Fig. 13; Table 2).
- Frequency: Two trials reported urinary frequency post-procedure^8,15^ (RR 0.46, 95% CI 0.23 to 0.94; RD − 0.44, 95% CI − 0.71 to − 0.17; I^2^ = 47%; *P* = 0.030; Fig. 14).
- Urgency: Two trials reported urinary urgency^8,15^ (RR 0.56, 95% CI 0.15 to 2.13; RD − 0.37, 95% CI − 0.90 to 0.17; I^2^ = 91%; *P* = 0.400; Fig. 15).

**Table 2:** Summary of findings for comparison between percutaneous nephrostomy and retrograde ureteral stenting for acute obstructive uropathy.

**Patient or population**: acute obstructive uropathy, **Setting**: emergency and day care, **Intervention**: PCN, **Comparison**: RUS						
Outcomes	Anticipated absolute effects* (95% CI)		Relative effect (95% CI)	No. of participants (studies)	Certainty of the evidence (GRADE)	Comments
	**Risk with RUS**	**Risk with PCN**				
Duration to WBC normalisation	The mean duration to WBC normalisation was **0**	MD **0.33 higher** (0.07 lower to 0.74 higher)	–	159 (2 RCTs)	⨁⨁⨁◯ MODERATE ^a^	Assumed risk calculated from the mean risk across the RUS group of the two included trials
Duration to defervescence	The mean duration to defervescence was **0**	MD **0.03 higher** (0.46 lower to 0.53 higher)	–	142 (2 RCTs)	⨁⨁⨁◯ MODERATE ^a^	Assumed risk calculated from the mean risk across the RUS group of the two included trials
Hospitalisation duration	The mean hospitalisation duration was **0**	MD **1.82 higher** (0.79 higher to 2.85 higher)	–	149 (2 RCTs)	⨁⨁⨁◯ MODERATE ^a^	Assumed risk calculated from the mean risk across the RUS group of the two included trials
QoL—mobility	31 per 100	**24 per 100** (8 to 76)	**RR 0.78** (0.25 to 2.48)	159 (3 RCTs)	⨁◯◯◯ VERY LOW ^a,b,c^	Assumed risk calculated from the mean risk across the RUS group of the three included trials
QoL—usual activity	34 per 100	**53 per 100** (19 to 100)	**RR 1.57** (0.55 to 4.53)	159 (3 RCTs)	⨁◯◯◯ VERY LOW ^a,b,c^	Assumed risk calculated from the mean risk across the RUS group of the three included trials
QoL—pain/discomfort	47 per 100	**46 per 100** (35 to 59)	**RR 0.97** (0.75 to 1.26)	159 (3 RCTs)	⨁⨁◯◯ LOW ^a,b^	Assumed risk calculated from the mean risk across the RUS group of the three included trials
Haematuria	38 per 100	**21 per 100** (14 to 32)	**RR 0.56** (0.37 to 0.85)	459 (4 RCTs)	⨁◯◯◯ VERY LOW ^a,b,c^	Assumed risk calculated from the mean risk across the RUS group of the four included trials

*The risk in the intervention group (and its 95% confidence interval) is based on the assumed risk in the comparison group and the **relative effect** of the intervention (and its 95% CI).

CI: Confidence interval; MD: Mean difference; RR: Risk ratio.

GRADE Working Group grades of evidence.

High certainty: We are very confident that the true effect lies close to that of the estimate of the effect.

Moderate certainty: We are moderately confident in the effect estimate: The true effect is likely to be close to the estimate of the effect, but there is a possibility that it is substantially different.

Low certainty: Our confidence in the effect estimate is limited: The true effect may be substantially different from the estimate of the effect.

Very low certainty: We have very little confidence in the effect estimate: The true effect is likely to be substantially different from the estimate of effect.

#### Secondary outcomes

**Figure 16:**
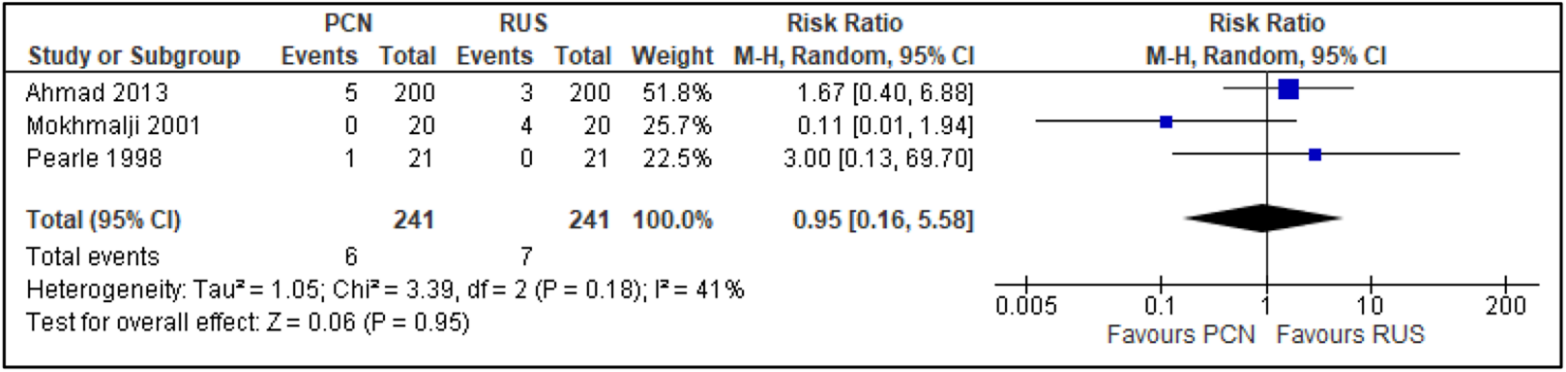
Comparison between PCN and RUS for outcome failure rate.

**Figure 17:**
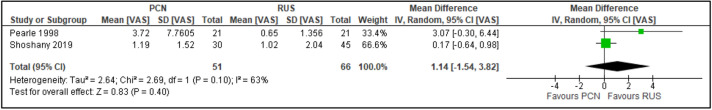
Comparison between PCN and RUS for outcome post-procedural pain (as measured by VAS).

1. Failure rates
  Three trials reported the number of failure rates for each procedure^10,29,30^. Two of these trials had a high risk of random sequence generation and allocation concealment^10,29^ (RR [95% CI] 0.95 [0.16 to 5.58]; RD [95% CI] − 0.02 [− 0.13 to 0.09]; I^2^ = 41%; *P* = 0.950; Fig. 16).
2. Post-procedural pain (VAS)
  Two trials reported quantitative assessment of post-procedural pain using VAS^14,30^ (two trials; 117 participants; MD [95% C1] 1.14 (− 1.54 to 3.82); I^2^ = 63%; *P* = 0.400; Fig. 17).
3. Analgesics use

**Figure 18:**
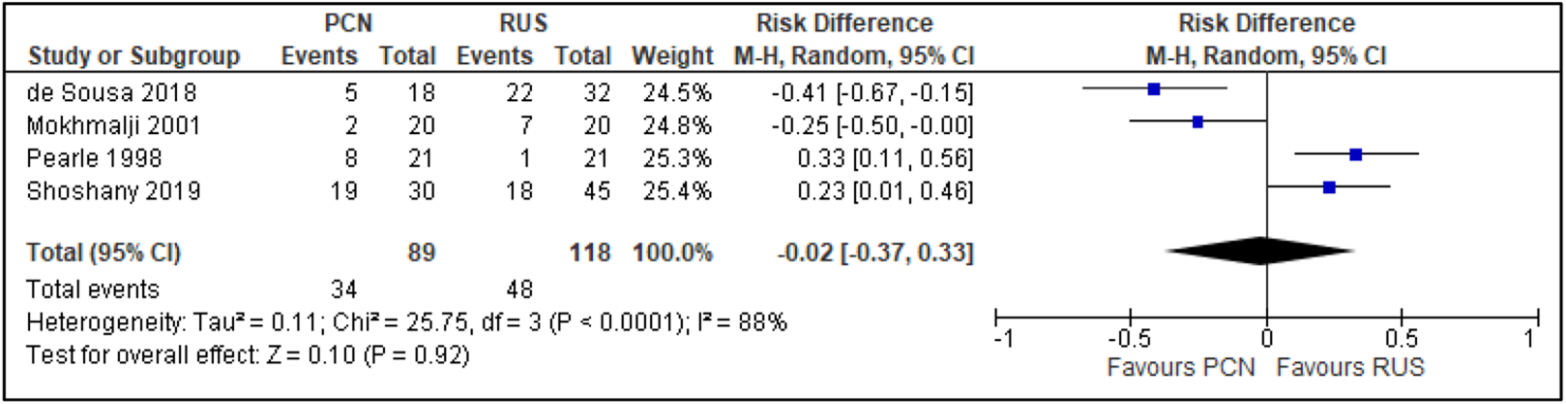
Comparison between PCN and RUS for outcome of analgesics use.

Four trials reported analgesics use to alleviate post-procedural pain^14,15,29,30^. Three trials had a high risk of random sequence generation and allocation concealment^14,15,29^ (RR [95% CI] 0.95 [0.31 to 2.93]; RD [95% CI] − 0.02 [− 0.37 to 0.33]; I^2^ = 81%; *P* = 0.920; Fig. 18).

## Discussion

This review aimed to compare the efficacy and safety of PCN and RUS for the treatment of acute obstructive uropathy based on available RCTs and CCTs. Consequently, only a few trials could be included in this meta-analysis for each outcome. PCN was associated with an increase in the hospitalisation duration compared with RUS. However, for outcomes of urinary-related symptoms, the findings were in favour of PCN, whereby more patients in the RUS group experienced haematuria and dysuria. These findings may affect the decision-making process of choosing these treatment options because although PCN may lead to longer hospitalisation duration, it has a reduced risk of causing a deleterious effect on the patient’s quality of life because of haematuria or dysuria after discharge to home. There were no differences regarding WBC normalisation duration, duration to defervescence, all five components of quality of life, urinary urgency and frequency, failure rates, quantitative assessment of pain post-procedure and analgesics use. Only one trial reported the comparison of the radiation dose received by patients undergoing these procedures; thus, the evidence is not conclusive. However, this is an important outcome that should be considered because exposure to radiation has substantial adverse effects on patients.

We conducted a thorough and extensive search of the databases and included seven trials that compared PCN and RUS. Stone disease constitutes the largest proportion of the obstruction cause in the included trials. Hence, the findings may not be applicable to other causes of obstructive uropathy, such as extrinsic obstruction due to malignancy or bladder abnormalities secondary to neurogenic bladder.

All included trials had a high risk of performance bias because of the surgical nature of the interventions, wherein patients are required to receive a detailed explanation and provide consent before the procedures are initiated, rendering the blinding of participants and personnel infeasible. However, this situation reflects the real-life scenario encountered in daily practice. Furthermore, most of the included trials also had a high risk of selection bias. Of these trials, five had a high risk of bias regarding random sequence generation, of which four also had a high risk of allocation concealment. In these trials, the decision to conduct either PCN or RUS was made solely on the preference of the performing surgeon based on experience or local protocol. A high risk of attrition bias was present in one trial, wherein patients who had an unsuccessful RUS were subjected to PCN.

We determined that the quality of evidence for the main comparisons ranged from moderate to very low. The quality of evidence was judged to be very low for quality of life outcomes regarding the impact of the procedures on mobility and usual activity of the patients and any occurrence of haematuria. This judgement was due to the high risk of performance, detection and selection bias of the relevant trials assessing these outcomes. We also detected a high heterogeneity among trials in these outcomes, which contributed to the reduced certainty of evidence in the findings.

We conducted an extensive literature search using multiple databases and by checking the reference lists of all related trials to minimise publication bias. For one trial, we were unable to analyse the data for the outcomes of duration to defervescence and hospitalisation duration because the data were presented in a non-usable format.

One systematic review involving six trials comparing the use of PCN with RUS for managing ureteral obstruction in benign and malignant aetiologies reported no difference regarding the time to clinical improvement in both PCN and RUS^31^. However, PCN was associated with a higher risk of post-procedure bacterial colonisation. Additionally, both procedures were also reported to have a high success rate. RUS insertion failure was reported to be amenable with PCN, but the contrary was not true. Regarding the quality of life assessment, this review reported that both procedures were associated with worsening quality of life. Nevertheless, no significant difference was reported.

In comparison with our review, most of these findings had an overall agreement with our data. However, a few exceptions were noted. The hospitalisation duration was significantly shorter in patients receiving RUS than in those receiving PCN, but PCN was superior regarding reported post-procedural haematuria and dysuria. Of the six trials in the previous review, four similar trials were included in our review^8,10,29,30^. The other two trials were excluded because of different reported outcomes of interest.

We recognised several limitations of our review. Firstly, there are limited number of trials conducted to compare the efficacy and safety of PCN versus RUS in the treatment of acute upper obstructive uropathy. Some of the outcomes could only be analysed based on data extracted from two studies. These include improvement in septic parameters, hospitalisation duration, urinary-related symptoms i.e. dysuria, frequency and urgency, and post-procedural pain. Secondly, there is a lack of RCTs conducted to compare these two types of intervention. We only found two RCTs to be included in our study. This is probably attributable to the urgent nature of the procedures rendering randomization difficult. Thirdly, all included trials contained high risk of performance bias. This was due to the surgical nature of the interventions which require written informed consent prior to the procedures. Hence, blinding of participants and personnel was not feasible.

In conclusion, we found no significant difference between PCN and RUS regarding improvement in septic parameters or quality of life post-procedure. Both methods are safe with high success rates. In clinical practice, we recommend the use of PCN rather than RUS, because the reduced quality of life due to haematuria and dysuria is much more devastating to patients over the long term. If further research is undertaken to compare PCN and RUS for the treatment of acute obstructive uropathy, we suggest the inclusion of radiation exposure and improvement of renal function as the main comparison.

## Methods

### Eligibility criteria

#### Types of studies

We included randomised controlled trials (RCTs) and controlled clinical trials (CCTs) comparing PCN and RUS.

#### Types of participants

We included patients with acute obstructive uropathy secondary to any cause who received PCN or RUS.

#### Types of interventions

We included studies on PCN conducted by a physician, using any size of nephrostomy tube, under ultrasound or fluoroscopy guidance with RUS as the comparison.

#### Types of outcome

We reviewed data on clinical success rates, described as improvement of septic parameters regarding duration to defervescence and normalisation of white blood cell count and hospitalisation duration. We evaluated information on quality of life and analysed data on urinary-related symptoms including haematuria, dysuria, frequency and urgency. For secondary outcomes, we reviewed information regarding failure rates of each procedure, post-procedural pain and analgesics use.

### Search strategies

We searched the Cochrane Central Register of Controlled Trials CENTRAL (latest issue), MEDLINE, EMBASE and CINAHL on 16 October 2019. A repeat search for trials that was performed on the 16 February 2021 yielded no additional trials that fulfil our inclusion criteria. The search strategy is described in Appendix 1. We adopted the search strategy for other databases. We restricted the publications to English language only.

We checked the reference list of identified RCTs, CCTs and review articles to find unpublished trials or trials not identified by electronic searches. We searched for ongoing trials through the World Health Organisation International Clinical Trials Registry Platform (http://www.who.int/ictrp/en/ and www.clinicaltrials.gov).

### Trial selection

We screened the titles and abstracts from the searches and obtained the full text of articles that appeared to meet the eligibility criteria or in instances wherein information was insufficient to determine study eligibility. We evaluated the eligibility of the trials independently and documented the reasons for exclusion. We resolved any disagreements between the review authors by discussion.

### Data extraction

Using a data extraction form, from each of the selected trials we extracted study settings, participant characteristics (age, sex and ethnicity), methodology (number of participants randomised and analysed), white blood cell count (WBC) normalisation duration, duration to defervescence, hospitalisation duration, quality of life based on questionnaires, urinary-related symptoms (e.g. haematuria, dysuria, urgency and frequency), technical success of each procedure to evaluate failure rate, pain score based on a visual analogue scale (VAS) and analgesics use.

### Risk of bias assessment

We assessed the risk of bias based on random sequence generation, allocation concealment, blinding of participants and personnel, blinding of outcome assessors, completeness of outcome data, selectivity of outcome reporting and other bias^32^. We resolved any disagreements by discussion.

### Grading quality of evidence

We assessed the quality of evidence for primary and secondary outcomes on the basis of the GRADE methodology for risk of bias, inconsistency, indirectness, imprecision and publication bias. The risk of bias was classified as very low, low, moderate or high^33^.

### Statistical analyses

We performed meta-analyses using Review Manager 5.3 software (RevMan 2014) and used the random effects model to pool data. We assessed the presence of heterogeneity in two steps. First, we assessed apparent heterogeneity at face value by comparing populations, settings, interventions and outcomes. Second, we assessed statistical heterogeneity using the I^2^ statistic^32^.

Thresholds for the interpretation of the I^2^ statistic can be misleading because the importance of inconsistency depends on several factors. We used the guide to the interpretation of heterogeneity as outlined: 0%–40% might not be important, 30%–60% may represent moderate heterogeneity, 50%–90% may represent substantial heterogeneity and 75%–100% would be considerable heterogeneity^32^.

We measured the treatment effect for dichotomous outcomes using risk ratios (RRs) and risk difference (RD) and for continuous outcomes using mean differences (MDs), both with 95% confidence intervals (CIs). No subgroup analysis was conducted in this meta-analysis.

We checked the included trials for the unit of analysis errors. These errors can occur when trials randomise participants to intervention or control groups in clusters but analyse the results using the total number of individual participants. We adjusted the results from trials showing the unit of analysis errors on the basis of the mean cluster size and intra-cluster correlation coefficient^32^.

We contacted the original trial authors to request missing or inadequately reported data. We conducted analyses of the available data when missing data were not available.

We conducted a sensitivity analysis to investigate the impact of the risk of bias for sequence generation and allocation concealment of included studies.

### Ethical standard

The manuscript does not contain original clinical studies.

## Supplementary Information

Supplementary Information.

## References

[1] Tseng TY, Stoller ML (2009). Obstructive uropathy. Clin. Geriatr. Med..

[2] Khan, A., *et al.* Epidemiology of chronic kidney disease in an adult Malaysian population (2014).

[3] Klahr S (1983). Pathophysiology of obstructive nephropathy. Kidney Int..

[4] Vaughan ED, Marion D, Poppas DP, Felsen D (2004). Pathophysiology of unilateral ureteral obstruction: studies from Charlottesville to New York. J. Urol..

[5] Lynch MF, Anson KM, Patel U (2006). Current opinion amongst radiologists and urologists in the UK on percutaneous nephrostomy and ureteric stent insertion for acute renal unobstruction: Results of a postal survey. BJU Int..

[6] Bansal T, Mehrotra P, Jayasena D, Okolo S, Yoong W, Govind A (2009). Obstructive nephropathy and chronic kidney disease secondary to uterine leiomyomas. Arch. Gynecol. Obstet..

[7] Pearle MS, Pierce HL, Miller GL, Summa JA, Mutz JM, Petty BA (1998). Optimal method of urgent decompression of the collecting system for obstruction and infection due to ureteral calculi. J. Urol..

[8] Joshi HB, Adams S, Obadeyi OO, Rao PN (2001). Nephrostomy tube or 'JJ' ureteric stent in ureteric obstruction: Assessment of patient perspectives using quality-of-life survey and utility analysis. Eur. Urol..

[9] Mokhmalji H, Braun PM, Martinez Portillo FJ, Siegsmund M, Alken P, Kohrmann KU (2001). Percutaneous nephrostomy versus ureteral stents for diversion of hydronephrosis caused by stones: A prospective, randomized clinical trial. J. Urol..

[10] Ahmad I, Saeed Pansota M, Tariq M, Shahzad Saleem M, Ali Tabassum S, Hussain A (2013). Comparison between double J (DJ) ureteral stenting and percutaneous nephrostomy (PCN) in obstructive uropathy. Pak. J. Med. Sci..

[11] Monsky WL, Molloy C, Jin B, Nolan T, Fernando D, Loh S (2013). Quality-of-life assessment after palliative interventions to manage malignant ureteral obstruction. Cardiovasc. Intervent. Radiol..

[12] Wang CJ, Hsu CS, Chen HW, Chang CH, Tsai PC (2016). Percutaneous nephrostomy versus ureteroscopic management of sepsis associated with ureteral stone impaction: A randomized controlled trial. Urolithiasis..

[13] de Sousa Morais, N., *et al.* Percutaneous nephrostomy vs ureteral stent for hydronephrosis secondary to ureteric calculi: impact on spontaneous stone passage and health-related quality of life-a prospective study. *Urolithiasis*. (2018). 10.1007/s00240-018-1078-2 30219938

[14] Shoshany O, Erlich T, Golan S, Kleinmann N, Baniel J, Rosenzweig B (2019). Ureteric stent versus percutaneous nephrostomy for acute ureteral obstruction—Clinical outcome and quality of life: A bi-center prospective study. BMC Urol..

[15] De Sousa Morais N, Pereira J, Mota P, Torres J, Cordeiro A, Anacleto S (2018). Which is the better urinary diversion regarding tolerability and quality of life-percutaneous nephrostomy vs ureteral stent: A prospective study. J. Endourol..

[16] Aravantinos E, Anagnostou T, Karatzas AD, Papakonstantinou W, Samarinas M, Melekos MD (2007). Percutaneous nephrostomy in patients with tumors of advanced stage: Treatment dilemmas and impact on clinical course and quality of life. J. Endourol..

[17] Emmert C, Rassler J, Kohler U (1997). Survival and quality of life after percutaneous nephrostomy for malignant ureteric obstruction in patients with terminal cervical cancer. Arch. Gynecol. Obstet..

[18] Flukes S, Hayne D, Kuan M, Wallace M, McMillan K, Rukin NJ (2015). Retrograde ureteric stent insertion in the management of infected obstructed kidneys. BJU Int..

[19] Gasparini M, Carroll P, Stoller M (1991). Palliative percutaneous and endoscopic urinary diversion for malignant ureteral obstruction. Urology.

[20] Ku JH, Lee SW, Jeon HG, Kim HH, Oh SJ (2004). Percutaneous nephrostomy versus indwelling ureteral stents in the management of extrinsic ureteral obstruction in advanced malignancies: Are there differences?. Urology.

[21] Netsch C, Becker B, Gross AJ (2016). Management of ureteral obstruction: Value of percutaneous nephrostomy and ureteral stents. Urologe.

[22] Pappas P, Stravodimos KG, Mitropoulos D, Kontopoulou C, Haramoglis S, Giannopoulou M (2000). Role of percutaneous urinary diversion in malignant and benign obstructive uropathy. J. Endourol..

[23] Gorelov, S., Zedan, F. & Startsev, V. The choice of urinary drainage in patients with ureteral calculi of solitary kidneys. Archivio italiano di urologia, andrologia : organo ufficiale [di] Societa italiana di ecografia urologica e nefrologica.**76**(2), 56–8 (2004). 15270414

[24] Hepperlen TW, Mardis HK, Kammandel H (1979). The pigtail ureteral stent in the cancer patient. J. Urol..

[25] Uppot RN, Uppot RN (2009). Emergent nephrostomy tube placement for acute urinary obstruction. Tech. Vasc. Interv. Radiol..

[26] Wilson JR, Urwin GH, Stower MJ (2005). The role of percutaneous nephrostomy in malignant ureteric obstruction. Ann. R. Coll. Surg. Engl..

[27] Zhao PT, Hoenig DM, Smith AD, Okeke Z (2016). A randomized controlled comparison of nephrostomy drainage vs ureteral stent following percutaneous nephrolithotomy using the Wisconsin stone QOL. J. Endourol..

[28] Pasiechnikov S, Buchok O, Sheremeta R, Banyra O (2015). Empirical treatment in patients with acute obstructive pyelonephritis. Infect. Disord. Drug Targets.

[29] Mokhmalji H, Braun PM, Martinez Portillo FJ, Siegsmund M, Alken P, Köhrmann KU (2001). Percutaneous nephrostomy versus ureteral stents for diversion of hydronephrosis caused by stones: A prospective, randomized clinical trial. J. Urol..

[30] Pearle MS, Pierce HL, Miller GL, Summa JA, Mutz JM, Petty BA (1998). Optimal method of urgent decompression of the collecting system for obstruction and infection due to ureteral calculi. J. Urol..

[31] Hsu L, Li H, Pucheril D, Hansen M, Littleton R, Peabody J (2016). Use of percutaneous nephrostomy and ureteral stenting in management of ureteral obstruction. World J. Nephrol..

[32] Higgins, T., *et al.* Cochrane handbook for systematic reviews of interventions version 6.0 (updated July 2019). *Cochrane*. (2019).

[33] Guyatt, G., *et al.* GRADE guidelines: 1. Introduction-GRADE evidence profiles and summary of findings tables. 2011(1878–5921 (Electronic)). 10.1016/j.jclinepi.2010.04.026 21195583

