# Supplementary material for: Percutaneous nephrostomy versus retrograde ureteral stenting for acute upper obstructive uropathy: a systematic review and meta-analysis
Source: Sci Rep. 2021 Mar 23;11:6613. doi: 10.1038/s41598-021-86136-y (PMC7988020; doi:10.1038/s41598-021-86136-y)
Supplement: Supplementary file 1 — Supplementary Information. [file 41598_2021_86136_MOESM1_ESM.pdf]

Appendix 1

MEDLINE

(((nephrostomy[Title/Abstract]) AND ("obstructive uropathy"[Title/Abstract] OR obstructi*[Title/Abstract] OR uropathy[Title/Abstract] OR hydronephrosis[Title/Abstract] OR sepsis[Title/Abstract] OR stone[Title/Abstract] OR calcul*[Title/Abstract] OR tumour[Title/Abstract] OR tumor[Title/Abstract] OR malignan*[Title/Abstract])) AND stent*[Title/Abstract]) AND ("quality of life"[Title/Abstract] OR complication*[Title/Abstract] OR pain[Title/Abstract] OR "urinary symptom*"[Title/Abstract])

CINAHL

AB nephrostomy AND AB (“obstructive uropathy” OR obstructi* OR hydronephrosis OR sepsis OR stone OR calcul* OR tum?r OR malignan*)

CENTRAL

(nephrostomy):ti,ab,kw AND ("obstructive uropathy" OR obstructi* OR uropathy OR hydronephrosis OR sepsis OR stone OR calcul* OR tum?r OR malignan*):ti,ab,kw AND (stent*):ti,ab,kw AND ("quality of life" OR complication* OR pain OR "urinary symptom*"):ti,ab,kw (Word variations have been searched)

EMBASE

'obstructive uropathy'/exp AND 'percutaneous nephrostomy'/exp AND 'Article'/it AND [adult]/lim AND 'Article'/it AND [adult]/lim AND ('clinical article'/de OR 'clinical trial'/de OR 'comparative study'/de OR 'controlled clinical trial'/de OR 'controlled study'/de OR 'human'/de OR 'major clinical study'/de OR 'prospective study'/de OR 'randomized controlled trial'/de OR 'retrospective study'/de)
